# Case Report: Rare vulvar myofibroblastoma

**DOI:** 10.3389/fonc.2026.1750550

**Published:** 2026-02-16

**Authors:** Jiaxin Miao, Shaoqin Sheng, Xiangqian Xu, Weiwei Qian, Yanying Nong

**Affiliations:** 1 The Fourth Clinical Medical College of Zhejiang Chinese Medical University, Hangzhou, China; 2 Department of Gynecology, Hangzhou Obstetrics and Gynecology Hospital, Hangzhou, China; 3 Clinical Medical College of Hangzhou Normal University, Hangzhou, China

**Keywords:** case report, clinicopathological features, diagnosis and treatment strategies, surgical management, vulvar myofibroblastoma

## Abstract

Vulvar Myofibroblastoma is a rare mesenchymal tissue tumor originating from myofibroblasts, with an unclear pathogenesis and biological behavior considered to be of uncertain malignant potential or low-grade malignancy. To investigate the clinical and pathological features of vulvar myofibroblastoma, a retrospective study was conducted on a rare case of vulvar myofibroblastoma admitted to Hangzhou Obstetrics and Gynecology Hospital. The patient was an elderly woman who presented with a 3-day history of a vulvar mass. Following initial examination, she underwent vulvar mass resection. Postoperative pathology suggested a myofibroblastic tumor with biological behavior of uncertain malignant potential or low-grade malignancy. As postoperative imaging evaluation indicated residual tumor, the patient subsequently underwent wide local excision of the vulva, which confirmed the diagnosis of vulvar myofibroblastoma. Short-term follow-up showed good recovery with no signs of recurrence or metastasis. In summary, for the diagnosis of rare vulvar myofibroblastoma, imaging examinations can help determine the tumor’s location, size, and relationship with surrounding tissues, but definitive diagnosis relies on histopathology. Treatment should aim for complete resection during the initial surgery. Given the uncertainty of its biological behavior, establishing a strict long-term follow-up mechanism is crucial for monitoring recurrence and ensuring the patient’s long-term prognosis.

## 1 Introduction

Vulvar mesenchymal tumors represent a heterogeneous group of neoplasms with diverse clinical behaviors and pathological features. Among these, myofibroblastomas are most frequently reported in the inguinal region. Although a small number of superficial vulvar myofibroblastomas have been described, deeply located primary vulvar myofibroblastomas remain exceedingly rare. Originating from myofibroblasts, these tumors are considered to have uncertain or low malignant potential. Due to their nonspecific clinical presentation, they are often overlooked or misdiagnosed as other entities such as angiomyofibroblastoma (AMF) or inflammatory myofibroblastic tumor (IMT). Definitive diagnosis relies heavily on histopathological examination and immunohistochemical profiling.

Here, we present a detailed clinicopathological study of a case of vulvar myofibroblastoma. The aim of this paper is to provide practitioners with actionable knowledge to enhance their understanding of this rare tumor.

## 2 Case description

**Figure 1:**
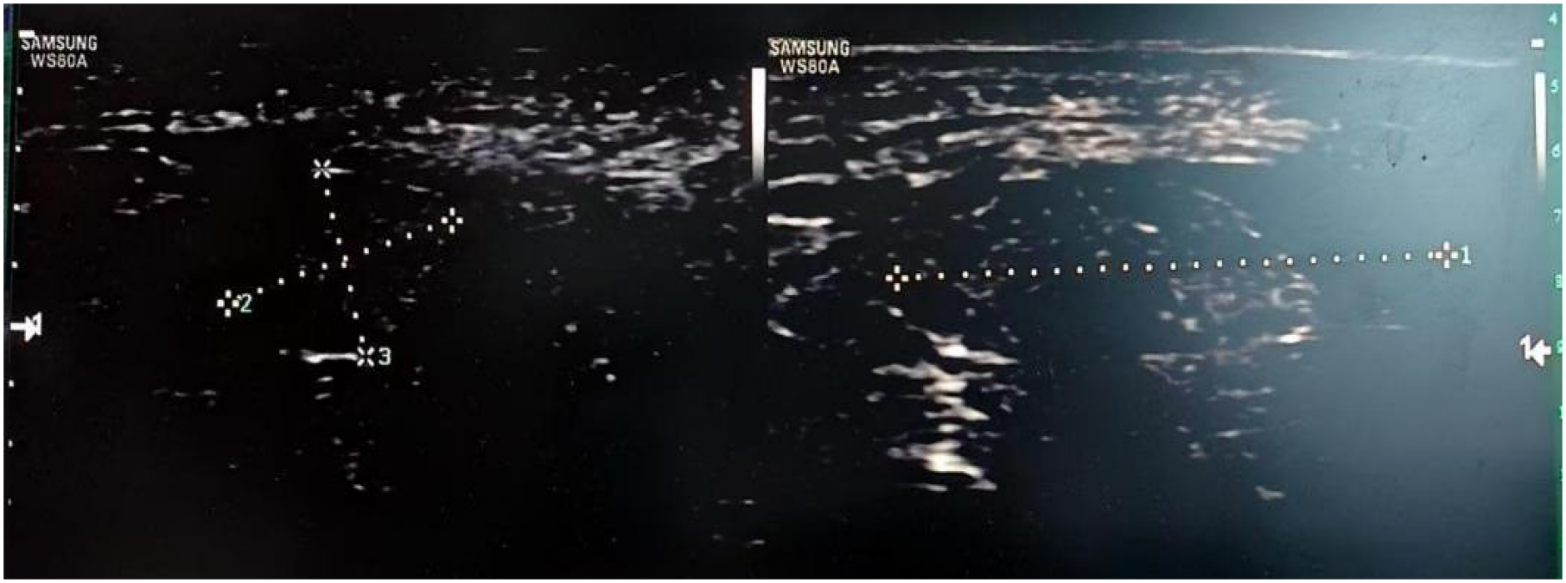
Preoperative transvaginal gynecologic ultrasound of the patient revealed that the right vulvar “mass” measured about 2.0*0.9*0.7 cm.

The 65-year-old married patient (P2) was admitted to the hospital on March 19, 2025, with a 3-day history of a vulvar mass. Three days prior to presentation, the patient discovered a vulvar mass on the right side of her vulva. The mass was about the size of a small walnut and was not painful to the touch. There was no redness, swelling, vaginal bleeding, abdominal pain, or bloating. There was no vaginal bleeding, abdominal pain, or distension. During gynecological examination, a hard, 2.0 x 1.5 cm mass with unclear borders was detected on the right side of the vulva. The vagina was patent, the cervix was light pink, and a polypoid mass measuring about 1.0 x 0.8 cm was seen at the cervical opening with no contact bleeding. No obvious abnormality was detected in the uterus or bilateral adnexal region. Considering the vulvar mass and cervical polyp, surgical treatment was recommended, and the patient was admitted to the hospital. She had a seven-year history of hypertension, controlled by oral antihypertensive medication, as well as a history of cesarean section. After admission, vaginal ultrasonography revealed a 1.3*0.6*0.4 cm long, tongue-shaped, weakly echogenic nodule in the cervical canal with clear borders that was protruding locally towards the cervical os. CDFI revealed a strip of blood flow signal. The right vulvar “mass” measured about 2.0*0.9*0.7 cm (**Figure 1**). It was uneven and slightly hypoechoic. The boundary was unclear. CDFI detected a blood flow signal. Arterial frequency harmonization was measured. RI was 0.67. Preliminary diagnosis: right vulvar mass and cervical polyp.

After admission, routine blood work, biochemistry, coagulation function, and tumor marker results were obtained, and no abnormalities were seen. Contraindications to surgery were ruled out, and vulvar mass excision and hysteroscopic electrocautery of cervical polyps were performed on March 20, 2025. During the operation, the vulvar mass measured about 2.0 x 0.9 x 0.7cm, pinkish-yellow in color, hard in texture, and poorly demarcated from the surrounding adipose tissue. The mass and some of the surrounding adipose tissues were removed with an electrocuter and quickly frozen. The frozen section revealed a spindle cell lesion/tumor. The spindle cells have mild morphology and do not show obvious heterogeneity or nuclear division. Therefore, mesenchymal lesions/tumors are considered. The nature of the mass cannot be clarified at this time. It is recommended that we wait for the postoperative routine pathology and immunohistochemistry results to make a definitive diagnosis and plan further treatment. Postoperative antibiotics were administered, and the incisions were treated with daily dressing changes.

**Figure 2:**
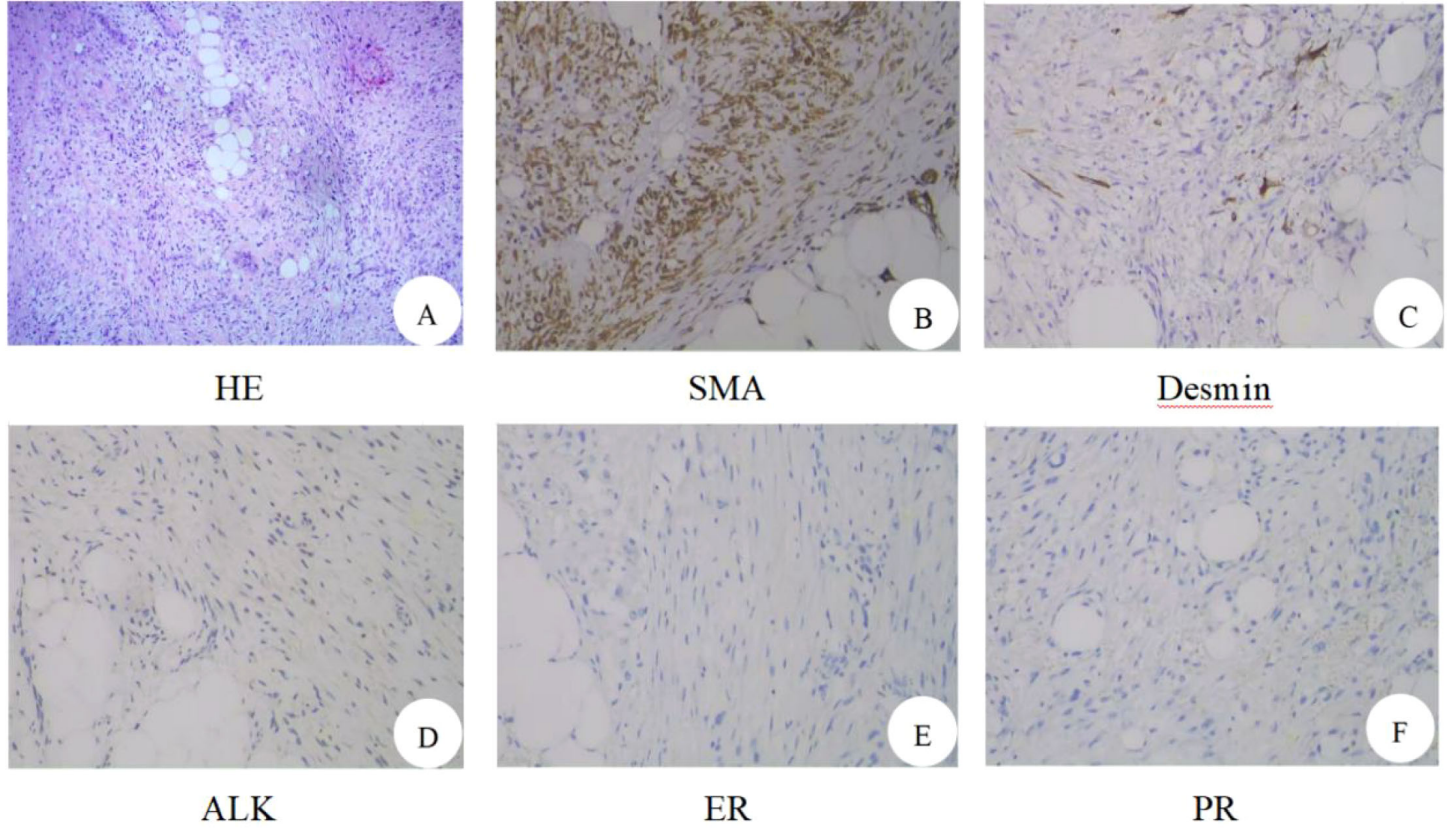
The result of hematoxylin-eosin staining: **(A)** Spindle cell lesion/tumor. Immunohistochemistry: **(B)** SMA, positive; **(C)** Desmin, focally positive; **(D)** ALK, negative; **(E)** ER, negative; **(F)** PR, negative.

**Figure 3:**
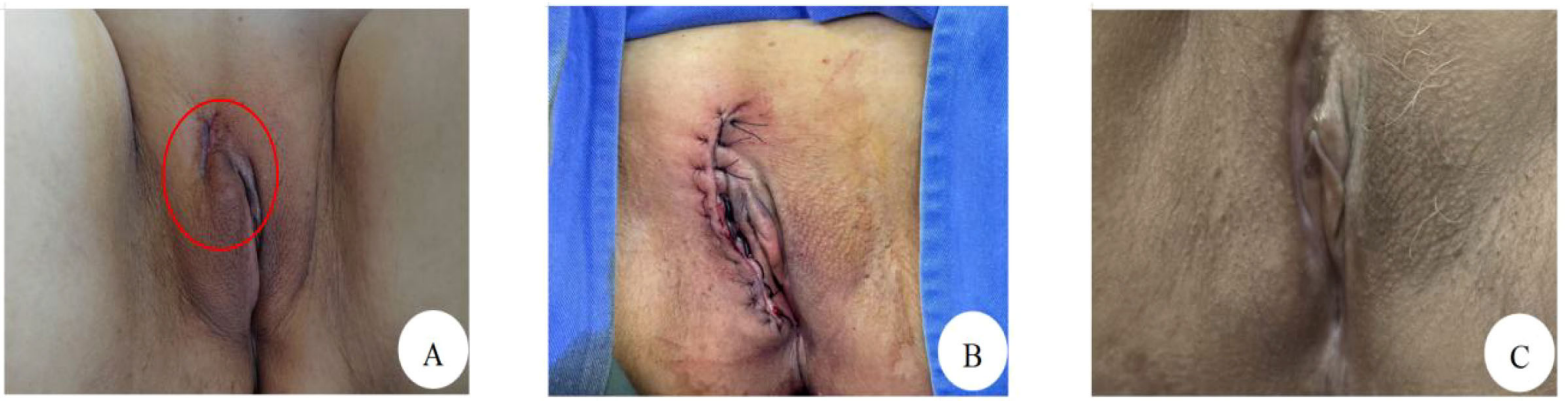
Physical examination. **(A)** After first surgery. **(B)** After second surgery. **(C)** Three months after surgery.

**Figure 4:**
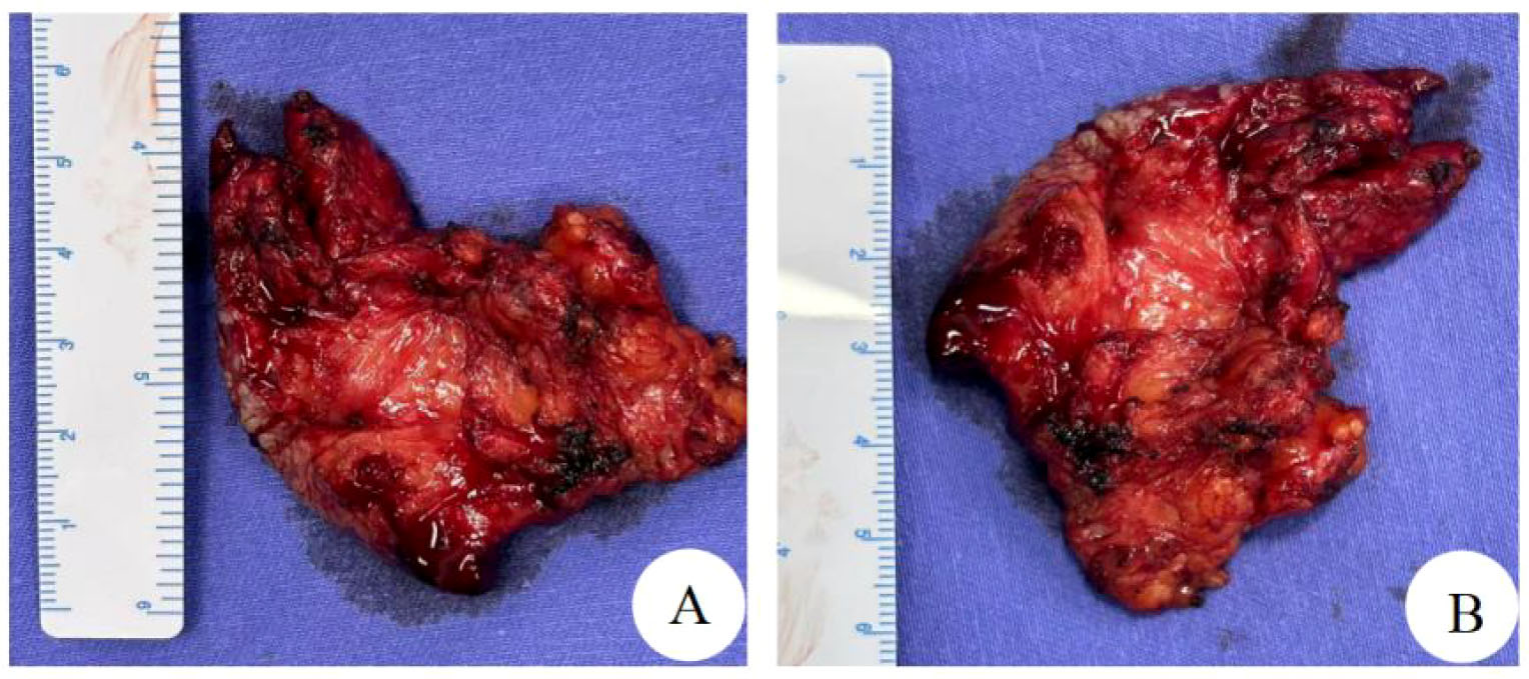
Gross findings **(A, B)**: Mass of fat interspersed with yellowish-pink fleshy tissue, poorly demarcated, with a hard texture.

On March 24, 2025, routine pathology suggested a spindle cell lesion/tumor in the vulvar mass. The spindle cell morphology was mild. The cells did not show significant anisotropy or nuclear fission. Spindle tumors were interspersed with growths in adipose tissue (**Figure 2A**). Combined with immunohistochemical findings, biological behavior was considered to be of undetermined or low malignant potential. Cervical polyp. Immunohistochemistry: focal desmin (+), SMA (+), CD34 vascular (+), beta-catenin with some intracellular plasma granules (+), S-100 (–), ALK (–), STAT6 (–), PR (–), ER (–), and Ki67 (+ with an average of 15% and 30%-40% in hot spots) (**Figures 2B–F**). On March 25, 2025, the patient’s vital signs were stable, and the incision was healing well. On examination, a hard mass about 1.0*0.5 cm in size with unclear borders, poor mobility, and no tenderness was palpable on the right side of the vulva (**Figure 3A**). On March 28, 2025, a pelvic MRI was performed, revealing altered morphologic signals in the cervical canal and anterior labial region. The two sides of the vulvar region had abnormal morphologic signals ranging from 3.2 cm x 7.2 cm x 2.1 cm with irregular morphology and uneven enhancement. There were no enlarged lymph nodes in the pelvis. On March 31, 2025, a wide local excision of the vulva was performed with complete excision of the mass 1.5 cm from the residual mass edge, deep to the fascia (**Figure 3B**). The excised tissues were sent for routine pathology. The postoperative anatomical specimen showed that the cut surface was interspersed with fat and yellowish-pink fleshy tissue with an unclear demarcation line and hard texture (**Figures 4A, B**). Postoperative vital signs were stable. The incision was not red, swollen, or oozing. The blood count was as follows: Leukocytes: 6.6*10^9/L, Neutrophils: 67.9%, Whole blood ultrasensitive C-reactive protein: 17.42 mg/L, and procalcitonin 0.12 ng/L. Postoperative conventional pathology suggested that the right vulvar lesion was a myofibroblastic tumor, and the biologic behavior suggested that it was of malignant potential. Undetermined or low malignancy, the tumor was close to the external and basal margins, and the inferior, internal, and cutaneous margins were negative. Desmin focal (+), SMA (+), S-100 (–), PR (–), ER (–), and Ki67 (+, mean 15%). Postoperative diagnosis: right vulvar myofibroblastoma, cervical polyp. She was discharged from the hospital. The patient was followed up at 1 month and 3 months after the operation (**Figure 3C**), recovered well, had no signs of recurrence or metastasis, and continued to be followed up.

## 3 Discussion

Vulvar Myofibroblastoma (VM) is a rare mesenchymal tissue tumor, originating from myofibroblasts, whose pathogenesis is unknown and whose biological behavior is considered to be of undetermined malignant potential or low malignancy. Myofibroblastoma occurs in 50% of cases in the inguinal/inguinal region, including the vulva/vagina, perineum, and scrotum (1), but cases originating in the vulva are extremely rare. Although a small number of superficial vulvar myofibroblastomas have been described (2), deeply located primary vulvar myofibroblastomas remain exceedingly rare.

Vulvar myofibroblastoma clinically manifests as a painless mass on the vulva. The tumor grows slowly and is often asymptomatic, and patients often consult a doctor only when the tumor has grown to a certain size. Therefore, it is crucial to increase awareness and early diagnosis of this disease. Imaging such as ultrasound and MRI can help determine the location, size, and relationship of the tumor to the surrounding tissues, but definitive diagnosis relies on histopathology and immunohistochemistry. The patient in this case presented with a painless vulvar mass. Although ultrasound suggested that the mass had poorly defined borders and was rich in blood flow, the mass was only considered to be a conventional vulvar mass in clinical practice, so simple excision of the mass was performed. Intraoperative freezing suggested a spindle cell lesion/tumor in the vulvar mass, and the spindle cell morphology was relatively benign, and the cells did not have any obvious anomalous shape or nuclear division image, so mesenchymal origin lesion/tumor was considered, and it was not possible to specify the nature of the mass at this time, and it was recommended to wait for Postoperative routine pathology and combined with immunohistochemistry results to clarify the diagnosis and further treatment. Postoperative routine pathology suggests low malignant potential. MRI has a unique value in assessing the extent of tumor infiltration and postoperative residuals. In this case, pelvic MRI before the second operation was used to comprehensively assess the relationship between the tumor and the surrounding tissues and found that: the cervical canal and anterior labial region had altered morphologic signal, and both were under-enhanced. The two sides of the vulva region had abnormal morphologic signal, ranging from about 3.2cmx7.2cmx2.1cm, with irregular morphology and uneven enhancement. No obvious enlarged lymph node shadow was seen in the pelvis. It provided a key basis for the adjustment of the surgical plan and fully embodied the core value of imaging in postoperative residual monitoring and staging.

It is important to note that vulvar myofibroblastoma may be easily confused with several mesenchymal tumors of the female genital tract in both clinical and pathological diagnosis, particularly vulvar angiomyofibroblastoma (AMF), inflammatory myofibroblastic tumor (IMT), and superficial cervicovaginal myofibroblastoma (SCVM). Clinically, vulvar myofibroblastoma may be misdiagnosed as vulvar AMF, which is also a rare mesenchymal tumor. Both present as slow-growing or painless masses without obvious symptoms, but typical AMF is characterized by a rich vascular network and spindle cell growth around blood vessels. Immunohistochemical staining shows that vulvar AMF generally expresses Vimentin and SMA, and >90% of cases are strongly positive for ER/PR, with a Ki-67 index of ≤10% (3). In contrast, inflammatory myofibroblastoma tumor is most commonly found in the uterus, followed by the cervix, ovaries, fallopian tubes, and broad ligaments in the female reproductive tract (4). IMT microscopically consists mainly of proliferating spindle fibroblasts/myofibroblasts in a fascicular or swirling arrangement, with a large inflammatory cellular infiltrate in the mesenchyme, mostly composed of mature plasma cells, lymphocytes, and eosinophils. Inflammatory myofibroblastoma (IMT) is histologically classified into three subtypes: the first is the mucoedematous type, with loosely arranged spindle cells separated by mucus-like mesenchyme, accompanied by neutrophilic/eosinophilic infiltration. The second is the fibrous histiocytoma-like type, with bundles of compact spindle cells, mixed with histiocytes, plasma cells, and lymphocytes. The third type is fibrotic, with elongated tumor cells scattered in dense collagenous interstitium with scattered eosinophils, lymphocytes, and plasma cell infiltration, and occasional calcification or ossification (5). Immunohistochemistry shows that inflammatory myofibroblastic fibroblastoma commonly expresses Vimentin, SMA, and partially CK or desmin, while CD117 and S-100 are negative, and the Ki-67 proliferation index is mostly <10%. ALK is a more characteristic marker, and studies have shown that it is expressed in at least 80% of IMTs of the female genital tract, if not more (6).

Additionally, superficial cervicovaginal myofibroblastoma (SCVM), also known as superficial myofibroblastoma of the lower female genital tract, shows overlapping histological and immunophenotypic features with vulvar myofibroblastoma, necessitating careful differential diagnosis. SCVM is a benign mesenchymal tumor of the lower female genital tract with a clearly benign biological behavior. Histologically, SCVM is usually confined to the subepithelial tissue and shows an expansile growth pattern, with a well-defined margin but without a true capsule. The tumor is composed predominantly of bland oval or spindle-shaped cells, with minimal cytological atypia and rare mitotic figures. The stroma is typically loose collagenous or myxoid in nature and may be accompanied by thin-walled vascular proliferation. Immunohistochemically, SCVM typically expresses Desmin and Vimentin, with ER and PR positivity in the vast majority of cases. The Ki-67 proliferation index is low (≤5%), and recurrence or metastasis is rare during follow-up (7). The microscopic presentation of the tumor in the vulva of our patient showed a mild morphology of spindle-shaped cells, the cells did not show significant anisotropy and nuclear division image, and the spindle-shaped tumor grew interspersed in the adipose tissue. Immunohistochemistry showed that the tumor cells expressed Desmin foci (+), SMA (+), CD34 vasculature (+), Beta-catenin some intracellular plasma granules (+), S-100 (–), ALK (–), STAT6 (–), PR (–), ER (–), and Ki67 (+, 15% on average, and 30%-40% in hot spots). Taken together, ER and PR negativity together with an elevated Ki-67 proliferation index argue against angiomyofibroblastoma (AMF), while negative ALK staining excludes inflammatory myofibroblastic tumor (IMT). Moreover, the deeply infiltrative growth pattern and increased proliferative activity are not consistent with the typical features of SCVM. In contrast to the clearly benign biological behavior of SCVM, the biological behavior of the present tumor is considered to be of uncertain malignant potential or low-grade malignancy. Abnormal β-catenin expression further supported the diagnosis of myofibroblastoma.

Complete surgical excision is the mainstay of treatment for vulvar myofibroblastoma. In this case, the first-time mass excision was performed, but postoperative MRI revealed residual lesions, and no obviously enlarged lymph node shadow was seen in the pelvis. Therefore, the second operation was performed with wide local excision to ensure negative margins (basal and outer margins were tight to the tumor), which is in line with the standard principles of management of low-grade malignancy. Long-term postoperative follow-up is a key component in the management of such rare tumors. In this case, no recurrence or metastasis was seen 3 months after surgery, suggesting the importance of early diagnosis and thorough surgery. However, due to the rarity of this tumor, its long-term biological behavior is unclear, and prolonged follow-up is needed to assess the long-term prognosis. The focus is on the primary site and regional lymph nodes. Therefore, regular follow-up is not only a means of efficacy assessment but also a guarantee of early intervention for recurrence.

## 4 Conclusion

In conclusion, vulvar myofibroblastoma is a clinically rare tumor of mesenchymal origin whose diagnosis and treatment still present many challenges. The definitive diagnosis of this disease requires a multidisciplinary evaluation of clinical manifestations, imaging features, pathomorphology, and immunohistochemical findings. Complete surgical resection is still the treatment of choice, and postoperative close long-term follow-up is required to monitor recurrence. Meanwhile, strengthening the multidisciplinary collaboration among gynecology, pathology, and imaging will help optimize the diagnosis and treatment process and further improve the prognosis of patients.

## Data Availability

The original contributions presented in the study are included in the article/supplementary material. Further inquiries can be directed to the corresponding author.
